# Comparison of CNN-Based Image Classification Approaches for Implementation of Low-Cost Multispectral Arcing Detection †

**DOI:** 10.3390/s26041268

**Published:** 2026-02-15

**Authors:** Elizabeth Piersall, Peter Fuhr

**Affiliations:** Oak Ridge National Laboratory, Electrification and Energy Infrastructures Division, Oak Ridge, TN 37830, USA; fuhrpl@ornl.gov

**Keywords:** machine learning, convolutional neural networks, multispectral sensing

## Abstract

Camera-based sensing has benefited in recent years from developments in machine learning data processing methods, as well as improved data collection options such as Unmanned Aerial Vehicles (UAV) mounted sensors. However, cost considerations, both for the initial purchase of sensors as well as updates, maintenance, or potential replacement if damaged, can limit adoption of more expensive sensing options for some applications. To evaluate more affordable options with less expensive, more available, and more easily replaceable hardware, we examine the use of machine learning-based image classification with custom datasets, utilizing deep learning based-image classification and the use of ensemble models for sensor fusion. Utilizing the same models for each camera to reduce technical overhead, we showed that for a very representative training dataset, camera-based detection can be successful for detection of electrical arcing. We also use multiple validation datasets, based on conditions expected to be of varying difficulty, to evaluate custom data. These results show that ensemble models of different data sources can mitigate risks from gaps in training data, though the system will be less redundant for those cases unless other precautions are taken. We found that with good quality custom datasets, data fusion models can be utilized without specialization in design to the specific cameras utilized, allowing for less specialized, more accessible equipment to be utilized as multispectral camera components. This approach can provide an alternative to expensive sensing equipment for applications in which lower-cost or more easily replaceable sensing equipment is desirable.

## 1. Introduction

Sensing applications have advanced greatly in recent years, due in part to technological developments in fields such as computation and Unmanned Aerial Vehicles (UAVs) as sensor platforms. However, while these developments increase potential applications for sensor deployment, many use cases also present the potential for damage, such as the potential for a UAV crash or other failure, or damage to remote sensors due to hazardous conditions. This risk can cause hesitance for the adoption of expensive sensor options, limiting applications. Camera-based sensing is an effective means of collecting data at a physical distance from the subject, and is a widely utilized approach for UAV and other remote platforms or high risk applications. However, risk factors can present potential restraints or cost limitations, which may be difficult to meet with off-the-shelf options, especially for smaller or more specialized applications. Commercially available sensor systems are often expensive, and multispectral sensor systems are often near or greater than the cost of many commercial UAV platforms. The inclusion of such a sensor could therefore significantly increase the initial purchase and replacement cost of damaged systems, and could limit usage or applications.

Developments in machine learning have the potential to expand data processing across many domains, including image classification. Deep learning methods in particular have been able to outperform other feature extraction approaches for image classification, and have shown value in a variety of remote sensing applications [1]. However, one of the most difficult aspects of the application of deep learning is obtaining and processing relevant datasets [2]. While the application of widely available datasets may be effective for some use cases, obtaining useful data for specific tasks may be difficult or impossible. In some of these cases, collection of custom data may be an alternative option. While not always a viable alternative due to restrictions on time, resources, or availability, custom data will be more relevant to the specific application, and ideally can be taken with the same sensors as the application. The sensor used can in some cases provide meaningful variation in data, for example, a specific camera can cause significant variation in image classification accuracy [3]. Even for more limited use cases, it may be difficult to capture data that fully covers the variation present within an application. For this context, the fusion of data from multiple sensors may be able to reduce the uncertainty of the overall model, allowing for a more limited, custom dataset to be applied more effectively over a variety of contexts.

In this paper, we present an evaluation of deep learning approaches utilizing custom datasets for a custom camera system, intended to be easily adaptable to varying and potentially hazardous applications. The application examined is the detection of electrical arcing, which fits the criteria for a use case where UAV or other remote sensing would be beneficial for human safety. Electrical arcing is a risk to a variety of industrial and infrastructure applications. Electrical utilities, for example, require timely awareness of arcing as a means of mitigating fire risk, as well as locating damaged equipment. Utilities often identify and locate a source of arcing through measurement of voltage and current anomalies, and use that information to guide human inspection for locating damaged equipment, increasingly aided by UAV data collection. Direct measurement of arcing that may be difficult to observe, such as from a damaged insulator and commercial solutions for corona measurement, can be prohibitively expensive to smaller utilities.

Our sensor uses a custom multispectral camera system, made up of RGB, ultraviolet, and thermal cameras for the measurement of a small source of arcing. In order to reduce cost risk, each of the cameras is a separate component to improve the overall repairability, with the approach to feature extraction and classification depending only on having data collected from the cameras, not the physical properties of the cameras themselves. This approach allows relatively easy replacement should a component become damaged and a direct replacement is unavailable.

This paper also evaluates the importance of quality of custom data with two versions of the dataset. The first combines all data collected, using a random subsection of the data for validation, resulting in training data that is likely very representative of the validation data. This is a common approach to evaluating machine learning models, and represents the ideal case when high quality, representative data can be collected. The second dataset instead separates out a section of data collected in a different physical location, so that no data from that exact location is included in the training dataset. This dataset is used to assess how the models perform on more novel conditions. This validation dataset is further separated into different levels of ambient light in order to assess the effect of unseen data on the model behavior relative to predicted difficulty and identify the likely source of difficulty. Both datasets are evaluated with ensemble models to assess improvement in results. The goal is to present an approach for a custom and customizable camera system, and to evaluate broadly applicable approaches to integrating lower cost, more available hardware for a specific application. As long as custom data can be collected, this information could be used to lower potential thresholds for sensor adoption.

This paper is organized as follows: The Relevant Literature Section provides a review of literature in multispectral sensing, arc detection, and ensemble models to provide context for the research done. The Materials and Methods Section describes the camera used, the process for collecting the data, and the process of machine learning. This includes the models chosen and the model parameters used. The Results Section is separated into Individual Results and Data Fusion. The Individual Cameras Section presents the results for the two datasets, and a comparison between the results of each. The Data Fusion results include results for the ensemble methods used, and a comparison of those methods with the addition of Gaussian noise. This is followed by a Discussions Section, and our Conclusions. This paper contributes an approach to implementing a multispectral sensor from low-cost components for the detection of electrical arcing, and an approach to building and evaluating a custom dataset for the implementation of that sensor. This includes evaluations on the importance of data quality and the behavior that can result from less representative datasets.

## 2. Relevant Literature

### 2.1. Multispectral Sensing

Multispectral data is electromagnetic data, often images or video, collected from the same source across multiple wavelength bands, which can include the visible, near-visible, infrared, and ultraviolet, depending on the application. The measurement of multiple spectral bands can add information to the spatial context provided by the visual range image, which can improve the ability of the system to determine chemical, physical, or thermal information about the object or area being observed. A widely used approach for the utilization of UAV-collected multispectral data is to collect overlapping spectral data using one sensor or several closely positioned sensors, the data from which can then be processed together. One example, ref. [4], utilized a Micasense RedEdge-M multispectral sensor for crop yield prediction. This sensor consisted of an RGB camera in addition to specialized RGB, red-edge, and NIR cameras, the images from each of which were stacked and used to calculate vegetative indexes as inputs for Random Forest, Gradient Boosting Regression, and Deep Neural Networks. The prediction outputs of these different models were then compared. The same Micasense sensor was used by [5] for the classification of plant communities with a Random Forest classifier used for cluster-based classification. In [6] data was captured with a DJI Phantom 4 Multispectral (P4M) sensor and used for the detection of weeds around citrus trees with Random Forest and KNN classifiers. A DJI Phantom 4 Multispectral sensor was also used by [7] for the monitoring of vegetation in a landslide area with classification done with a support vector machine with a linear kernel.

UAV-based thermal imaging was utilized by [8] for the measurement of roosting fruit bat populations, using a Zenmuse XT 19 mm radiometric thermal camera, which gave researchers access to environments that would otherwise have been difficult to traverse. Another use of UAV data collection in a challenging environment is shown in [9], in which an Unmanned Surface Vehicle (USV) carried multispectral imagery sensors used for monitoring ocean environments. The data for this study was collected with two sensors, a 20-megapixel CMOS sensor for RGB data and MicaSense RedEdge-MX multispectral camera which included visual and near-infrared data, used for shallow water bathymetry. A similar application using visual-only cameras, collected with a UAV with the default DJI Phantom 4 4K camera, was used to monitor soil erosion in [10] to collect a reliable dataset to assess vegetation and soil cover.

A review of hyperspectral imaging for agriculture and forestry was done in [11], in which hyperspectral imaging describes the measurement of many, at times hundreds, of narrow spectral bands. The paper includes descriptions of commercially available hyperspectral sensors for UAV use, and discusses that the development of such sensors requires coordination between the sensor manufacturer, the UAV manufacturer, and an additional party to provide integration between those systems. These requirements can limit the utility of commercially available sensors for UAV applications through higher price points, limited supply, and compatibility issues with other UAV platforms.

These access limitations have led to custom implementations of multispectral and hyperspectral sensors for a variety of applications. A modular, low-cost prototype multispectral camera is described in [12], which utilizes a fixed camera with a filter wheel, allowing a single camera with consistent spatial information to capture different spectral wavelengths. The generation of a custom dataset using separate, side by side cameras was done by [13], intended for applications in pedestrian detection for autonomous vehicles. This experiment used a Logicool, HD PRO WEBCAM C920R RGB camera, a Nippon Avionics InfReC R500 far-infrared (FIR), a Nippon Avionics InfReC H8000 mid-infrared (MIR), and a Xenics, Xeva-1.7-320 NIR. The data from each of these cameras was used as input for a separate You Only Look Once (YOLO) classification models, the outputs of which were combined with an ensemble process by comparing the predicted bounding boxes from each output.

### 2.2. Arc Detection

The example application examined in this paper is the optical detection of an electrical arc, which is applicable to safety inspections in applications including equipment maintenance and fire prevention. Arcs generate both light and heat, making multispectral cameras a potentially effective approach for identification. The detection of electrical arcs is a field of interest to many applications, including those suited to camera-based monitoring. Camera-based detection is used as a step in a hierarchical algorithm in [14]. A microphone array is used to identify a characteristic sound by an electrical arc, and is used to signal the system that one has occurred. The system then estimates the distance to the fault and verifies the fault location by checking for high temperatures on the insulator with a thermal camera. In [15] a charged coupled device (CCD) camera is used with a high frame rate to capture an image of a low voltage electrical arc, with an edge detection algorithm to identify it.

Detection of arcs in a pantograph-catenary system on a train is discussed in [16]. Arcs occur when the contact wire does not stay attached to the pantograph, or due to wear or damage to components. A vision-based detection method for such trains is described in [17], which detects arcs from a video recording the pantograph. The use of a photomultiplier tube to detect ultraviolet light for arc detection of a pantograph is described in [18]. In [19], data from an infrared camera is stored and analyzed to detect overheating or arcing to assist with preventative maintenance. A computer vision-based approach for pantograph-catenary arc detection based on Otsu’s method is described in [20] using a collected time series of grayscale images.

The value of ultraviolet sensors in arc detection is discussed in [21], which characterizes spectra measured from electrical arcs used in urban rail, measuring from 200 to 1100 nm. Parts of these spectra are located in the UV spectrum in the solar-blind region, indicating the UV light could be measured without too much interference from sunlight, and could therefore be useful data for arc detection. Another application described in [22] uses an ultraviolet sensor, which measures the presence of ultraviolet light as a signal, to detect partial discharges on ceramic insulators. The resulting spectrograms were classified with k-nearest neighbor (kNN), Naive Bayes, Decision Tree and SVM classifiers. Another study, ref. [23], used images of a porcelain insulator taken with a FILIN6 UV imager with flashover, and used AlexNet, a convolutional neural network, to classify the results and evaluate their use as a flashover detector. The use of a combination of ultraviolet and visual cameras was also described in [24] as a low-cost detector for coronas caused by partial electrical discharges in aeronautic environments.

### 2.3. Ensemble Models

Ensemble learning describes the use of more than one model to process data, where the results are combined into a single output with the intention of improving the quality of the prediction. Ensemble models are a type of fusion model composed of multiple component models, inputting the same data into models with different architectures, or related but different data into similar models, and then fusing the outputs of those models’ decisions into a single result.

Fusion models are often described as fusing information at the data, feature, or decision level. An example of a feature fusion model is used by [25], with multiple embedded CNN models taking preprocessed subsections of data and extracting features for further analysis, which are then concatenated, with additional fully connected layers used for the identification of a source camera used for the image. The embedded CNNs included AlexNet and ResNet18 models, with better results found in the fused system than those individual models. A feature fusion system was used by [26] to combine 3D LiDAR data with 2D RGB camera data, with ResNet as the backbone for feature extraction. A similar problem was addressed by [27], which utilized CNNs both to extract features from LiDAR and RGB images, and for object detection of the fused data. Computational units of decision fusion were used in [28], in which outputs from decision fusion models were concatenated as part of a hierarchical model for image classification. The paper found better results with decision fusion for CNN outputs than feature fusion in this context. In [29], a system for gait recognition is described, combining feature extraction from distance and speed data, classification with a kernel extreme learning machine and decision fusion of the classification outputs from multiple frames of data.

An ensemble to combine model architectures is used by [30], which classifies medical images according to the results from four different medical image datasets according to the combined results from different models, a process referred to as stacking. This was used in combination with other techniques, such as augmenting, performing rotations or other modifications to training data to reduce overfitting, and bagging, training the multiple models from randomly chosen selections from the training dataset.

Ensemble methods are discussed in the survey [31] in addition to other classification approaches for hyperspectral data. The ensemble methods described include the use of a support vector machine (SVM) and random feature selection, the integration of different CNN-based classifiers, and the use of separate CNN and deep residual classifiers, as well as different approaches to the integration of spatial and spectral data provided by hyperspectral sensors. In [32], a custom CNN architecture was used for the base classifiers, concatenating the results to models augmented with Gaussian noise before fusing the results. In [33], a CNN ensemble was used to address small sample size in hyperspectral data, using a pixel-pair feature (PPF) algorithm integrated with an ensemble CNN classifier followed by majority voting.

An example of the use of an ensemble model to address uncertainty from lighting conditions is discussed in [34], which describes the use of image classification in a robotic grasping system for an industrial application. A random cropping ensemble neural network (RCE-NN), which uses randomly cropped subsections of an image of the material to be classified, used an ensemble of CNN-based feature extractors made of AlexNet_bn, Inception, VGGNet, and ResNet, and was combined with a weighted majority voting algorithm.

For multispectral data, ensemble models can be used to achieve various advantages over individual models. Indices calculated from multispectral data can be used as inputs to multiple models, which can then be combined to improve the accuracy of the output. This approach includes examples such as [35], which examined the use of bagging, or combining predictions of models after prediction through approaches, such as a majority vote, and boosting, a sequential approach to combining predictors in order to improve the errors of previous predictors. These approaches were applied to both multispectral and hyperspectral datasets. Boosting was also used by [36] for the estimation of plant chlorophyll content with data from RedEdge-M MicaSense to improve regression estimates done by KNN and SVM. The ensemble approach of stacking, or combining the outputs of constituent models into a metamodel, was used by [37] for the classification of mangrove species. An ensemble of CNNs for hyperspectral data, as well as visible and LiDAR data, is described in [38], which used extracted features as inputs for CNNs for both the hyperspectral and LiDAR + RGB data, which were fused using Weighted Majority Voting and Behavior Knowledge Space. Adaptive weighting approaches which utilize generative adversarial networks for the fusion of the spatial and spectral information of hyperspectral data are compared to other hyperspectral and multispectral image fusions. Overlapping spectral data were compared for additional applications as well. In [39], adaptive weighting is analyzed for different image resolutions, and ref. [40] covered image classification of land cover features.

## 3. Materials and Methods

Three cameras were used for this combined sensor: a visual, an ultraviolet, and a thermal camera, all of which can be connected by USB to any computer or microcontroller as needed for adaptability, and none of which cost more than $350 USD at the time of purchase. The visual camera is an ELP 48 Megapixel USB camera, manufactured by Shenzhen Ailipu Technology Co., Ltd., Shenzhen, China. This camera has a resolution of 3840 × 2160 at 30 frames per second, and the lens is 1/2.3” with an 80 degree field of view. The ultraviolet camera LDP LLC XNiteUSB2S-MUV: USB 2.0 Megapixel HD Monochrome camera, manufactured by Llewellyn Data Processing, Carlstadt, NJ, USA. This camera has a resolution of 1920 × 1080 at 30 fps, and the lens is 3.6 mm with a 170 degree field of view. This is a low-cost ultraviolet option with a glass lens, limiting the spectral range to 365–380 nm, as the capacity to measure shorter wavelengths requires a significantly more expensive quartz lens, increasing the cost of the overall camera by around $1000 USD. The thermal camera is a USB FLIR Lepton 3.5 thermal camera, manufactured by Teledyne Flir LLC, Wilsonville, OR, USA, used with a PureThermal Mini Pro JST-SR webcam, manufactured by GroupGets, Reno, NV, USA. The FLIR Lepton is a radiometric capable longwave infrared camera with a spectral range of 8000 to 14,000 nm, with a resolution of 160 × 120, and a radiometric accuracy of +/− °C, and a frame rate of 8.7 Hz.

**Figure 1:**
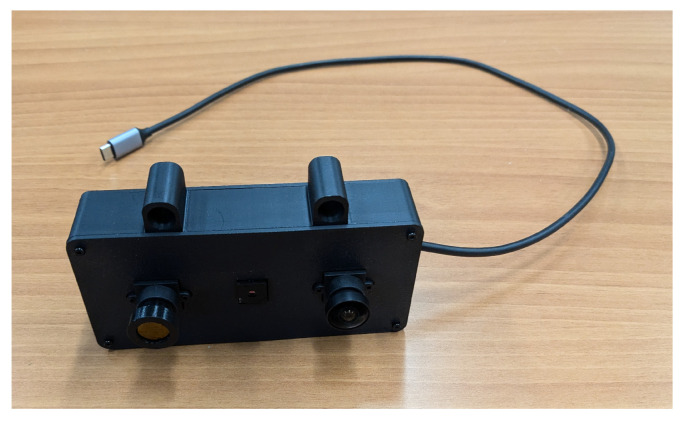
Experimental cameras in 3D-printed mount.

Data capture was done with an OpenCV script to collect images from all three cameras together. The script connects each of the cameras, then saves an image from each camera on key press. Each image is both labeled and timestamped at the time of collection. After cleaning the data to remove images containing anomalies, such as misalignments between the source and the cameras, or malfunctions in either the source or camera, the images were sorted into datasets and given labels both to coordinate the data points automatically by model, and verified manually if needed. An example label is thermal_2024-12-12T15:37:06-04_frame_000_00000533.png, following the format: [camera]_[original time stamp]_[frame during collection]_[unique id]. The images are also sorted into directories by camera type. The first three fields, camera type, the original time stamp, and the numbered frame taken during data collection, are included in the final image label to allow the images to be manually referenced and verified if needed. The final number is unique per camera, with one number for each image taken together. This number is used by the ensemble models to automatically correlate the connected images. These labels are present in the dataset, which has been made publicly available. The spacial relationship between the camera images was maintained throughout data collection by fixing the cameras in a 3D-printed mount like the one shown in Figure 1; the order and spacing of which was consistent throughout the data collection process.

The goal of this combined sensor was to investigate a camera-based arc detection approach that would have relatively low barriers to adoption, and so would be accessible for the inspection of electrical equipment in contexts where physical risk may present barriers to use, such as low-cost UAV platforms, or in hostile environments. The combined camera system described in this paper was less than $800 USD at the time of purchase, and could be reconfigured with different USB cameras if needed with minimal changes to the design, as a lack of repair or replacement options can also be a limitation to adoption for users. The challenge presented by this custom approach is the potential variability in camera design, resolution, and behavior. Feature extraction approaches usable with one camera option would not necessarily behave in the same way for another, even for a similar spectral response. This paper will focus on examining a design option for a CNN-based approach for image classification, utilizing a custom training data for both feature extraction and classification.

**Figure 2:**
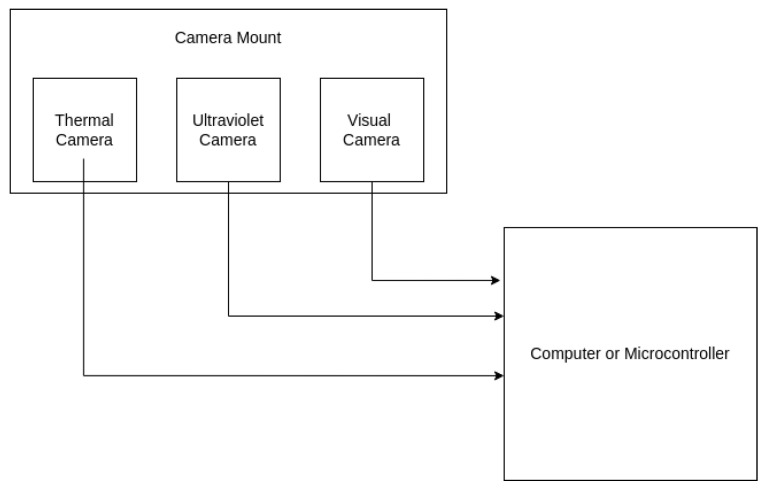
Diagram of cameras during data collection.

**Figure 3:**
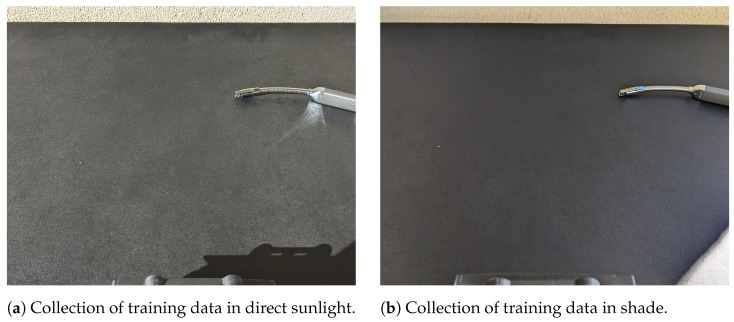
Examples of collection of training data with different levels of ambient light.

The data was collected with each camera connected to a laptop as shown in Figure 2. Two sets of data were collected, both across several days in order to capture varying levels of natural light, as shown in Figure 3. The cameras were connected to a laptop with the arc source, a Zippo Rechargeable Candle Lighter, manufactured by Zippo Manufacturing Company, Bradford, PA, USA, held in front at a distance varying between 6 and 18 inches from the camera mount. Two classes were collected, labeled ‘off’ and ‘on’, in which the lighter was either off, with no visible arc, or on, with a visible arc. The data was stored in directories with these labels, allowing for supervised machine learning to be performed. The cameras were allowed to adjust their exposure time according to the light conditions based on default settings, as would be the expected behavior in a field application. Additional details regarding the data collection and cameras can be found in [41]. The conditions chosen for this data collection most closely mimic cameras that are either fixed or pan-tilt-zoom, monitoring equipment relatively close to the camera mount, and other factors that may need to be considered for custom data collection for specific applications may include UAV vibration, greater variation in physical offset, or variation in equipment temperature.

All data were collected in the same general physical location, with variation added due to the positioning and angle of the cameras and arc source, as well as the time of day and weather conditions. The training dataset was collected with a variety of different outdoor backgrounds, including the walls of a building from varying angles, as well as greenery and sky. Of this dataset, approximately 45% of the data was taken in sunlight, 44% in indirect sunlight due to shade or cloudy conditions, and 11% taken in low-light conditions near dusk. The original percentage of low-light data was smaller, closer to 5%, but experimentation showed that moderately lit data from cloudy weather and low-light data near dusk behaved differently, and so more data collected near dusk was added.

The second dataset collected was a validation dataset, made up of four smaller datasets, each collected entirely at one time in order to contain a fairly consistent level of natural light. Of these validation datasets, two were collected in sunny to partially cloudy conditions and one in cloudy conditions, all collected near midday, and one in low-light conditions, collected near dusk. These datasets are less varied than the training dataset, with all four validation datasets collected in the same specific outdoor location, against a wall of a building, with no rotation of the cameras throughout collection. While the arc source was still moved throughout the frame of the three cameras in order to add variety to the data, the cameras were kept in the same location and facing the same direction throughout all validation data, limiting the significant differences between each validation dataset to the ambient light conditions. The goal of this process was to create consistency across the validation data, so that the variation in classification accuracy due to the light levels specifically could be assessed. The details of the number of data points per class, where one data point consists of one image taken from each of the three cameras, are shown in Table 1.

For this research, the training and validation datasets were collected to have a close to even balance between the classes. While a balanced dataset may be difficult to capture from real world data for a dataset like arcing, this dataset is intended to represent a case for which the conditions measured can be replicated under controlled conditions, and therefore a balanced dataset can be generated. The number of images from each camera is the same, as they were taken together, and are coordinated by number in order to facilitate coordination for the ensemble models. The small difference in number between the classes was due to data cleaning, and additional images taken to make sure there would be an adequate number of good quality images in the final dataset.

**Figure 4:**
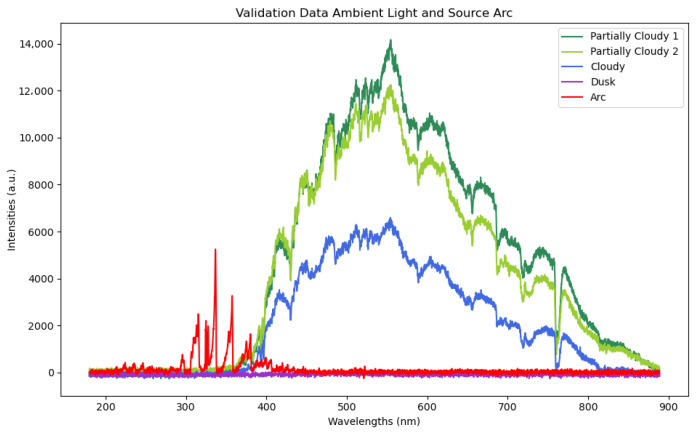
Data from Ocean Optics spectrometer from the validation data and the electrical arc, combined into one graph. Overlapping data between the arc and ambient light represents wavelengths at which ambient light levels may obscure light emitted from the arc source.

**Figure 5:**
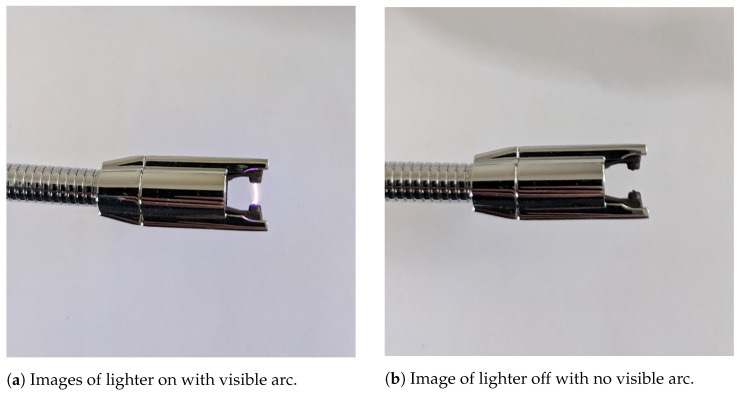
Images of the Zippo Rechargeable Candle Lighter used for data collection on and off, manufactured by Zippo Manufacturing Company, Bradford, PA, USA.

**Figure 6:**
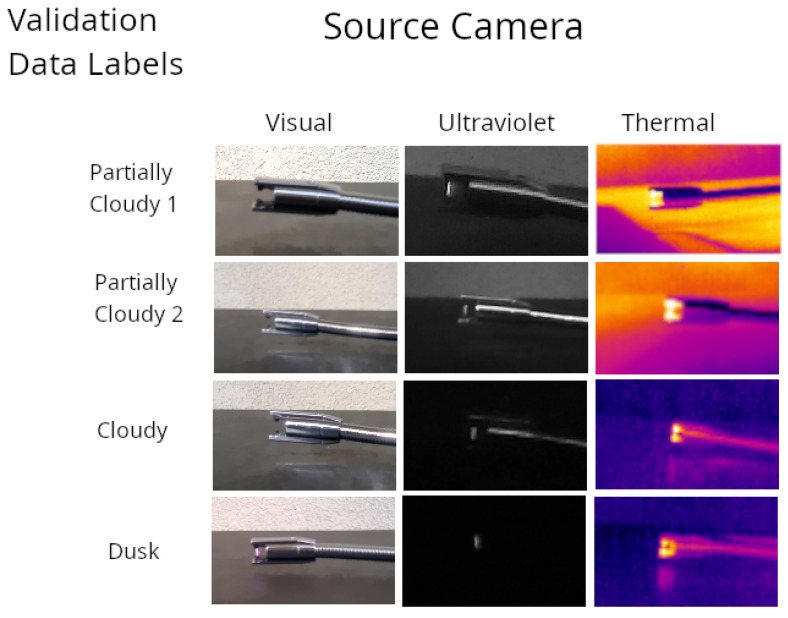
Example images taken from the four validation datasets. The columns represent the three cameras used for data collection, and the rows represent the four validation datasets shown in Figure 4, as well as in the results to follow. These images all contain an active arc, and demonstrate the differences in arc visible from each camera relative to levels of ambient light across the validation datasets. The images are representative of images taken from each camera, with RGB for the visual camera, monochrome for the ultraviolet camera, and the default Ironbow palette for the thermal camera, with hotter objects represented with lighter and warmer colors. Lighter used manufactured by Zippo Manufacturing Company, Bradford, PA, USA.

The ambient light conditions at the time of collection for each of the four validation datasets, as well as the spectra of the electrical arc from the lighter, were collected with a USB connected Ocean Optics spectrometer, manufactured by Ocean Optics, Orlando, FL, USA and are included in Figure 4. This diagram shows the light available from the arcing source compared to the ambient light present during each data collection. The ultraviolet cameras with its range of 365–380 nm have the most capacity to capture this light, though capturing more of this range would require a more sensitive, and therefore more expensive and less accessible, ultraviolet camera. The thermal camera instead captures radiant thermal information from the heat generated by the lighter, and was included to reduce the impact of ambient light on sensing capability. Examples of the lighter on and off taken with a separate camera are shown in Figure 5, and with the experimental cameras in Figure 6.

A table of the ambient conditions of the validation data is shown in Table 2. At the location and time of year recorded, sunrise was at approximately 7:38 a.m. and sunset was at approximately 5:23 p.m., with the location against a building to the north and open to the south, providing the most sun mid-afternoon. Three of the four validation datasets (Partially Cloudy 1, Partially Cloudy 2, Cloudy) were collected near midday, around 12:00 p.m., on sequential days with different weather conditions, and are labeled according to the weather conditions, as determined by sight and verified by a NOAA forecast for the area. The fourth (Dusk) was collected late in the day shortly after 5:00 p.m., just before sunset.

The results of the image classification of the models tested will first be shown for each camera separately, followed by a comparison of ensemble approaches. As part of evaluating the ability to use custom data to train a custom set of cameras, two approaches to dataset collection will be compared. The first, labeled Full Dataset, will combine all of the data, including the validation dataset, into one shuffled dataset, and training and validation subsets will be randomly selected. The second, labeled Validation Dataset, will maintain the separation from dataset collection, and show the results across the different levels of ambient light. This step was included to assess how the approaches perform on data with more or less variation from the training dataset. While data points are not repeated in the training and validation data in the Full Dataset, the training data will likely contain very similar images to the validation data, resulting in a likely easier validation dataset for the trained models. The Validation Dataset is intended to have enough variation from the training data, and will also provide more specific results relative to different levels of ambient light.

### 3.1. Machine Learning

Machine learning has been implemented in many data analysis contexts for applications such as the identification of features, or an identifiable section of a system [42], and patterns that may, under other conditions, require an expert to identify. For image classification, the data is collected or obtained, formatted, and preprocessed prior to classification by a model. Models are often either regression-based, define a relationship between data and future values, classification-based, or identify and separate data into distinct class labels [43]. The quality of the dataset used for training, such as the representativeness of the dataset or the balance of the classes, will impact the quality of the trained model. An imbalanced dataset is one where there are different numbers of samples of one class than another, such as for a rare event that is difficult to record. When an imbalanced dataset is used to train a model, the model will likely prioritize the samples with more data [44].

Neural networks utilize artificial neurons, data structures with value-based activations and weights that can be set with training, connected into layered networks. During training for supervised learning using labeled data, these structures extract features from input data by passing the data through the network, determining the error or loss from the expected label, and backpropagating to calculate changes to the weights. This process is performed over many interactions of the training set, called epochs. A many-layered neural network is called a deep neural network, and has shown advantages over other machine learning approaches when identifying patterns in complex data in various contexts [45]. Convolutional neural networks, or CNNs, are neural networks which contain convolutional layers for feature extraction. Each neuron in a CNN is only connected to a small number of neurons in the previous layer, reducing parameters when compared to fully connected layers. These networks also frequently contain pooling layers, which can take advantage of local image correlation to reduce the amount of data while retaining useful information. Many model architectures for CNNs have been developed, published, and utilized in recent years. The models tested with this dataset are described below.

AlexNet was published in 2012 and was developed to classify images in the ImageNet dataset, consisting of 1000 different classes. As was needed for Imagenet, AlexNet was designed with the intention of creating a network with a large learning capacity. This model is made up of eight layers, which are five convolutional and max pooling layers and three fully connected layers [46]. The output of the neurons uses a nonlinear activation function known as Rectified Linear Units (ReLUs) characterized by the function f(x)=max(0,x) as opposed to alternative functions such as f(x)=tanh(x), which allows faster training times. This architecture includes max pooling layers, which summarize adjacent neurons and can help reduce overfitting, and dropout layers, which set neurons to zero with a probability of 0.5, and force the trained network to become more robust.

VGGNet was published in 2015, and was also developed to classify the ImageNet dataset, with the goal of improving image classification accuracy over other CNNs. VGGNet uses small convolutional filters in layers, separated by max pooling layers with eleven to eighteen weight layers, and ending with three fully connected layers [47].

ResNet was published in 2015 and was originally tested on several datasets, including PASCAL and COCO. This architecture represents an approach to addressing the problem of vanishing and exploding gradients that occurs with deeper networks, and can prevent very large convolutional networks from training effectively. ResNet approaches this problem by using residual layers as an attempt to optimize feature mapping within the network [48].

These architectures chosen are widely used, well-studied models which perform well on a variety of image classification tasks, while also training and running efficiently without the need for more advanced hardware. This choice was intended to allow the models to work over a variety of inputs, and to adapt with minimal modification. The three models also allow for enough variation in performance to assess the performance of the datasets across various conditions. Given the relatively straightforward nature of the task being examined in this research, binary classification of a source either being on or off under fairly controlled conditions, the smaller versions of these model architectures were used. While VGGNet16 and ResNet32 were tested with preliminary versions of this dataset, the results described below used VGGNet11, ResNet18, and AlexNet, which was adequate for comparison with this dataset. More complex architectures may be warranted for more complex applications.

### 3.2. Model Parameters

Training configurations included a starting learning rate of 0.001, which was decreased by a factor of 0.1 every 7 epochs. Cross-entropy loss was used as the criterion, and stochastic gradient descent was used as the optimizer, with a momentum of 0.9. The PyTorch library, version 2.4.0 was used for the machine learning, and the PyTorch implementations of AlexNet, Resnet18, and VGGNet11 were used for the models. Preprocessing was done to format the images to a consistent size, which was necessary for the transfer learning. Additionally, augmenting was used to add variety to the training dataset, with random horizontal and vertical rotations, and randomized changes to the brightness, contrast, saturation, and hue of the images of all three cameras during the training process. The augmenting approach leveraged existing transform functions within the PyTorch library, specifically RandomHorizontalFlip, RandomVerticalFlip, and ColorJitter. RandomHorizontalFlip and RandomVerticalFlip invert the image across its vertical or horizontal axis using a probability of 0.5. ColorJitter randomly adjusts four parameters: brightness, contrast, saturation, and hue. These values were selected uniformly using the parameters: max(0, 1 − brightness), 1 + brightness, max(0, 1 − contrast), 1 + contrast, max(0, 1 − saturation), 1 + saturation, −hue, hue.

### 3.3. Transfer Learning

Transfer learning describes the process of transferring knowledge of models trained on a source domain to a task in a target domain, where the task of the source domain is related to the task of the target domain [49]. In the case of the following experiments, both the source and task domains are intended for the task of image classification. The pretrained weights of the source domain were trained on the ImageNet dataset, as provided by the PyTorch library. Models were trained both with and without the use of transfer learning as a means of comparing the behavior of the classification model architectures, the results of which are shown in the following sections.

The transfer learning used in this paper utilized pretrained weights through the PyTorch library. The default weights were used, which at the time of this work were the weights with the label IMAGENET1K_V2, trained on the ImageNet dataset.

### 3.4. Metrics

A common metric for displaying the outcome of a classification model is the accuracy, for which the outcome of classification can be described in the following terms: True Positive (TP) and True Negative (TN) for correct classification, and False Positive (FP) and False Negative (FN) for incorrect classification [50]. The accuracy describes the ratio of correct predictions to total instances in the form of the following equation [51]:

(1) $$Accuracy=\frac{TP+TN}{TP+FP+TN+FN}$$

The effectiveness of the accuracy as a classification metric can be dependent on how well balanced the dataset is, in that if there are far more of one class than another it can skew the results and obscure the smaller class if it is performing poorly. This can be examined with other metrics, such as the precision, the portion of a positive class that is correctly identified as positives, or the recall, the portion of identified positives which were actually positives. The F1 score, another common metric, is the harmonic mean of the precision and the recall. This metric is intended to favor models with similar Precision and Recall scores, and provide more weight to the effects of smaller classes in the dataset. The equation for the F1 metric is:

(2) $$F1\;\mathrm{Score}=\frac{2*TP}{2*TP+FP+FN}$$

The choice of metric can vary based on what aspects of the model and data need to be additionally validated. A well balanced dataset, one with approximately the same number of values per class, may be adequately described by the accuracy, while an imbalanced dataset may require different metrics such as the F1 score to effectively represent the performance across all classes. While accuracy will primarily be used in this work, the F1 score for the individual models that will be included verifies consistency with the accuracy results.

## 4. Results

### 4.1. Individual Cameras

The results for image classification are first examined for each of the cameras individually, using the three different model architectures described above, with and without the use of transfer learning. This step is included to generate a baseline of classification capability for data from each camera across the different model architectures, and to identify the effects of model design choices for each camera and across the variations in datasets.

#### 4.1.1. Full Dataset

The full dataset combines the collected training and validation data to provide a baseline for the separate validation results. This full dataset has a total of 2917 images in the ‘on’ class and 2900 images in the ‘off’ class, with small differences between class numbers from data cleaning, which removed around 10–20 images from each class due to alignment issues, or a malfunction with the arc source. To make sure there was no variation in behavior in this dataset, and to evaluate all the data for consistency, 5-fold cross-validation was performed for each of the models compared.

Cross-validation is an approach to the training and validation of models in which the data is divided into sections, with different sections used for training and validation to prevent overfitting and to compare the performance on different data. K-fold cross-validation is the process of dividing a dataset into *k* near-equally sized segments, with k−1 segments used to train the model, and the hold-out segment used for testing [52], which is then repeated until all folds have been rotated through as the testing hold-out. The difference between this approach and a simpler 80/20 train/test split is that all of the data was shuffled into the sections used for the folds and was rotated over, so that over the folds all data has been used both as training and hold-out validation. The results from this full dataset are intended to both act as proof of concept for the ability of the CNN models to detect the presence of arcing for using our test source, as well as to provide a point of reference for the more challenging Validation Dataset tests described in the following section.

The results of this cross-validation of the full dataset are shown in Table 3, where the average of the 5-fold accuracy and F1 scores are shown for three different models, AlexNet, ResNet18, and VGGNet11, for images taken with each of the three cameras, both with and without the use of transfer learning. Overall the metrics were very good, especially for the ResNet18 and VGGNet11 models, and in this case, the results with and without transfer learning were similar. Given the F1 results show no significant difference in behavior from the accuracy results, the full database will be considered well balanced for the remainder of the paper, and only accuracy used for simplicity. The results from this dataset show that this data can be used to effectively train models for classification of arcing, assuming the training data is representative of the validation data.

#### 4.1.2. Validation Dataset

For this dataset, the validation data was collected in a different physical location than the training dataset, and at a different angle relative to incident light. These conditions were held consistently throughout the validation data, with the only significant differences between the validation being the level of ambient light. These differences between the training and validation datasets were intended to make the dataset more difficult for the classification task, and as a result, more effective at identifying the relative difficulty of the varying levels of ambient light present in the four validation datasets for data from the different cameras. In an ideal application, the training dataset would be fully representative, which, as shown in the previous section, was very successful in identifying arcs across the conditions tested. However, due to the difficulty of assessing all possible conditions, how representational a dataset is in all relevant cases may be difficult to assess. These results aim to assess the capabilities of CNN model architectures to address this potential limitation.

The same model architectures, with and without transfer learning, were also compared for the validation dataset. This data was trained with an 80/20 train/test split selected randomly from the training dataset; in this case, the validation dataset was kept entirely separate. The validation results, shown in Table 4, show these accuracy results. A final column including the averaged results of all four validation datasets is included to help represent overall patterns.

Without very similar data from the training set, both the ultraviolet and visual cameras show notable reductions in accuracy with brighter ambient light, shown with the two partially cloudy validation datasets. This was likely due in part to the different angle of light changing the appearance of light in the image in ways that were different from the training dataset. In this case, the more challenging data can also be used to compare different model parameters, and how well they can perform against conditions outside of class balances that may not be evenly represented in the training data, something that could be difficult to fully avoid in some types of application data.

The models using transfer learning have overall higher accuracies for all three cameras. Comparing the models, ResNet18 had the highest accuracies in most cases, with VGGNet11 as a close second, with better performance in some cases. Comparing the cameras, the RGB visual range camera was the most impacted by the levels of ambient light, with the lowest accuracies in bright light. Ultraviolet was affected by light levels as well, though still had greater than 90% accuracies for ResNet18 and VGGNet11 in low-light cases. The thermal camera was not affected by the light levels, and was the only camera to have comparable accuracies to the results with the full datasets, implying that the thermal training data remained more representative of the validation results. This result can be explained by referencing the spectrometer results in Figure 4. Sunlight contains significant electromagnetic radiation in the ranges of visual and ultraviolet of the spectrum, with the spectrometer data in this example showing the majority of light between 400 and 850 nm. A light source detected optically in the presence of sunlight will become more difficult to distinguish. An ultraviolet source will be less significantly overwhelmed, though the limited range of a low-cost ultraviolet camera will reduce the effect. Thermal radiation, which would be measured by these longwave thermal cameras in the 8000 to 14,000 nm range, would be reasonably expected to be less impacted by this limitation due to a lack of solar radiation in that part of the spectrum, which is represented in the results.

#### 4.1.3. Result Comparison

**Figure 7:**
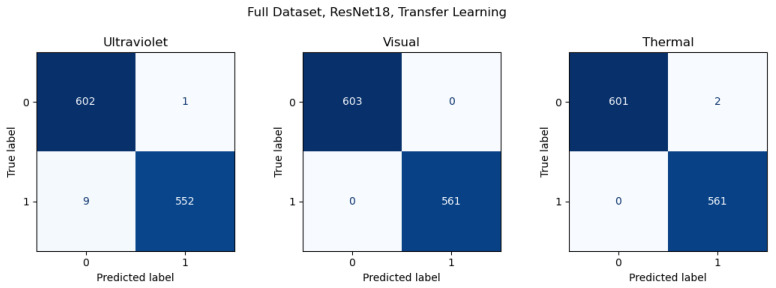
Confusion matrices for Full Dataset results for transfer learning with ResNet18. Darker colors represent a greater number of values.

**Figure 8:**
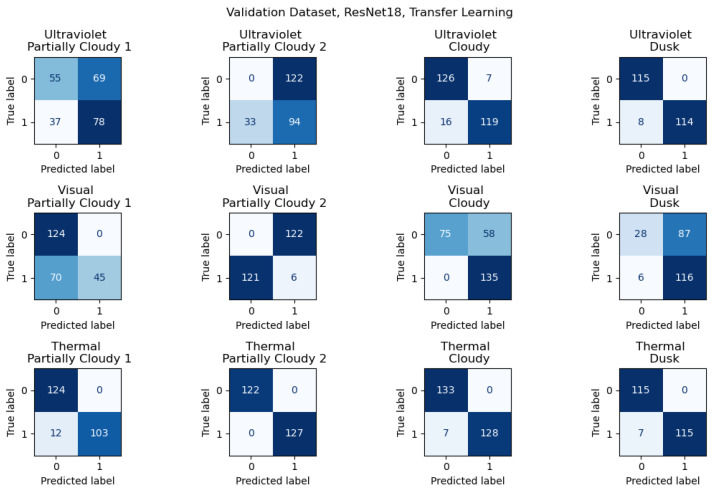
Confusion matrices for Validation Dataset results for transfer learning with ResNet18. Darker colors represent a greater number of values.

A comparison of the results from the Full Dataset and Validation Dataset illustrates the importance of adequately representative data, especially in the case of more difficult environments. The same images are used in both datasets, but the Validation Dataset separates images from a specific location for validation. The results illustrate the impact of less representative data, especially in the context of more difficult environmental conditions. To further represent this conclusion, confusion matrices are included for samples of both results, using transfer learning with ResNet18 results for consistency. The results for the Full Dataset are shown in Figure 7, and the results for the Validation Dataset in Figure 8.

For the Validation Dataset, the results from validation sets with greater levels of sunlight had significantly lower levels of accuracy for both the Visual and Ultraviolet camera data. This pattern is not seen in the Full Dataset, despite it being different organizations of the same data. The primary difference between the two datasets is the validation data from the Validation Dataset is kept entirely separate, with no representative data from the location of its validation datasets, including in the training data, while the Full Dataset is fully shuffled, with the training and validation data selected randomly. The locations are similar between the data, leaving the primary variable as the angle of incident sunlight. As described above, the training data is taken at a variety of angles relative to sunlight, but none with the light directly behind the camera, while the validation data was collected with the sun behind the camera.

**Figure 9:**
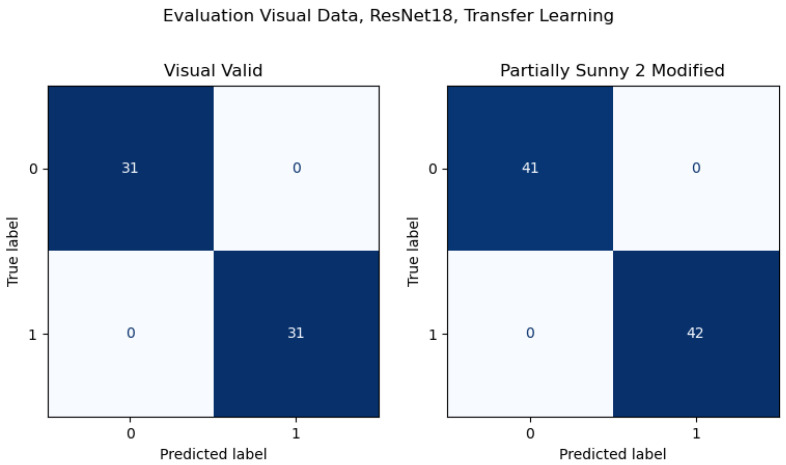
Confusion matrices for the evaluation datasets for the Visual data, for transfer learning with ResNet18. Both represent the classification ability of models trained on very representative data to successfully classify samples with bright ambient light. Darker colors represent a greater number of values.

To test this suspected cause more directly, a third dataset was made from the Visual data in the Validation Dataset, manually removing samples taken in direct sunlight, and adding in samples from the original Partially Sunny 2 validation dataset. This modified training dataset was used to train a ResNet18 model and tested against two validation datasets made from the sunny data removed from the training data and the remaining data in Partially Sunny 2 that was not added to the training dataset, which have been labeled Visual Valid and Partially Sunny 2 Modified respectively. There was no overlapped data between the new training and validation datasets, but the context of the new validation datasets was more fully represented. These models were trained with a random 80/20 split within the training data. These results over validation for this model are shown in Figure 9. The high accuracy of these results is consistent with the Full Dataset results, demonstrating that visual data with bright ambient light can be classified successfully if trained on representative data. However, as is shown by the Validation Dataset results, these more difficult cases are also more dependent on high quality data.

### 4.2. Data Fusion

A comparison of how the use of simple ensemble approaches for data fusion affects and can potentially improve the results of the validation dataset is presented in this section. The same preprocessing was done for all three cameras as for the individual cameras. As was the case for the individual camera results, augmenting was used to add variety to the training dataset, with custom functions used to apply the same transformation to each image in a set. Transfer learning was used for the models in all cases based on the consistent improvement seen in the individual results for the validation dataset.

#### 4.2.1. Majority Vote

Two approaches to a majority vote were compared, which combined the separate models used in the previous sections into one result. The first was a simple majority vote, described by:

(3) $$V=\underset{j}{arg max}\sum_{i=1}^{M}c_{i}^{j}$$

**Figure 10:**
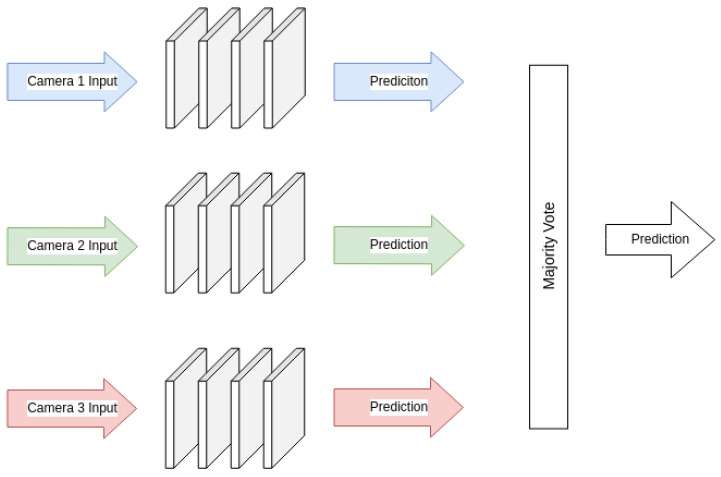
Diagram of majority vote ensemble with three inputs.

This vote determines the prediction from the input models has a straightforward form of decision fusion, outputting the final prediction result. A diagram of this ensemble is shown in Figure 10. The majority vote results using models of each of the three models from the previous section, AlexNet, ResNet18, and VGGNet11, are included in the results. The second approach is a weighted majority vote, which is similar to the approach used in [53], which used results from a test dataset to determine the weights for each model. All models, including all three cameras from all three model architectures, were combined into a final result. This weighted result is described by:

(4) $$V=\underset{j}{arg max}\sum_{i=1}^{M}w_{i}c_{i}^{j}$$

As with the individual results, the ensemble results are shown for both the Full and Validation datasets. The Full Dataset simple majority vote results are shown in Table 5, and represent very high accuracies that are consistent with the cross-validation results from this dataset. The output accuracies of of the less representative data for implementations of a majority vote are shown in Table 6, with the results for the three separate models included for the simple majority vote. All models are included within the weighted majority vote result, which is included for the Validation Dataset to compare its potential to additionally improve the results. Overall, the majority vote improved the results for most cases in this dataset. The most difficult of the validation datasets, Partially Cloudy 2, was the primary exception, showing that while improvements can be made with this approach, data quality still presents significant limitations.

#### 4.2.2. Combined CNN

**Figure 11:**
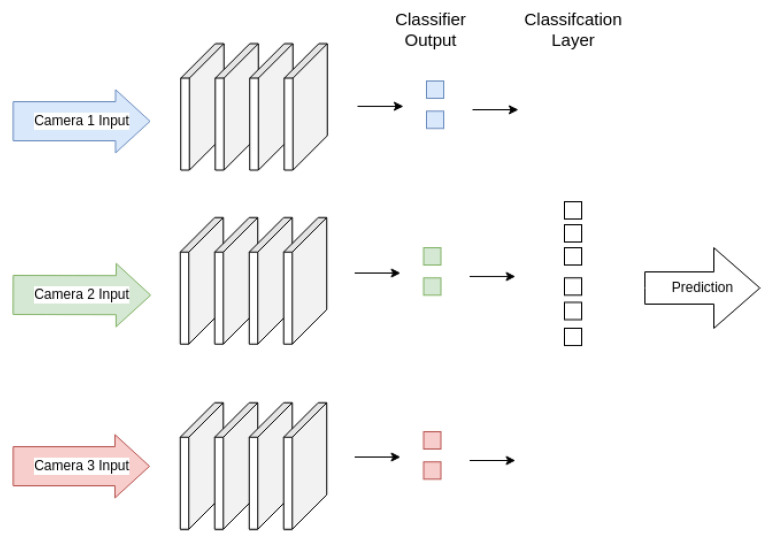
Diagram of combined CNN ensemble for three input models.

The other ensemble model compared is a combined CNN, which takes inputs of each of the models used for image classification, and combines the result in a final, single output. PyTorch implementations of AlexNet, ResNet18, and VGGNet11 were used again to build the combined CNNs, which retained most of the original architecture of models, but removed the final classification layer and replaced it with a single combined layer. The final layers in a CNN are used for classification, with the early layers of the model extracting features of different degrees of complexity depending on their placement within the model [54]. The goal of this combined model is to retain the feature extraction layers for each of the models for each camera, but to train the full system of models with one classification layer and one combined output, as each model within this dataset will have two outputs, with one for each class. The final layer takes six inputs when using data from three cameras. A diagram of these models is shown in Figure 11. These models were trained with the same parameters as the separate models, using custom classes for randomized image augmentation across all three input images to add variety to the dataset and improve training.

The results for these models overall had consistently good results for both datasets, with the trained weights able to accurately classify results even with variation absent in the training data. The results of this combined model are shown for the Full Dataset in Table 7 and the Validation Dataset results are shown in Table 8.

### 4.3. Addition of Camera Noise

A final test was included to evaluate the resilience of these models to an additional source of noise affecting the input data on the ensembles. This was included to compare how model fusion, whether directly applied through the majority vote or trained with data in the combined CNNs, was resilient to an obstruction in one of the cameras. Gaussian noise was added to each of the cameras, lowering the image quality to the extent that the image classification was nearly random. In an application, this sort of distortion could be caused by physical damage or obstruction to a camera, or due to corruption of saved or transmitted data. Distortion to this extent would represent a problem with the camera system that would ideally be detected through other means or prevented with maintenance, and so a complete failure of a camera would represent one of the worst cases the camera system could be expected to operate under, and lower performance would be expected.

Gaussian noise was added at the data transform stage of the model; the built-in Pytorch function GaussianNoise was used to add noise to the input tensor for a provided mean and sigma. Detailed results for the inclusion of significant noise for the majority vote with noise added are included in Table 9, and for the combined CNNs in Table 10. What qualified as significant noise was determined with experimentation of the values that consistently reduced all accuracy to around 0.5, a random guess, and for these results was set at sigma = 50.

Predictably, reducing the data quality of one of the camera inputs also reduced the classification accuracy in most cases. An exception can be seen for the unweighted majority voting for ultraviolet and visual noise for the validation dataset Partially Cloudy 2. This is likely due to this particular dataset often being misidentified as the wrong category, likely due to reflected light being misinterpreted by the model. As the very noisy data is instead close to a random guess, that is what is reflected in the results. Another trend is that the weighted models, both the manually weighted majority vote and the CNN with trained weights showed a stronger drop in accuracy when the thermal data is noisy than with the other inputs. For the majority vote, the fact that higher weights were placed on that data is easy to verify, and so the result was expected. While the specific weights cannot be directly determined for the CNN, given that the behavior of the models relative to noise being added to different cameras is the same, it is likely to be the same cause of behavior, indicating the combined CNN is placing higher weights on the thermal inputs as well. Again, this shows the limitation of ensemble methods to overcome limitations in data quality, as well as the potential for more complex models to hide potentially important data biases without careful evaluation.

**Figure 12:**
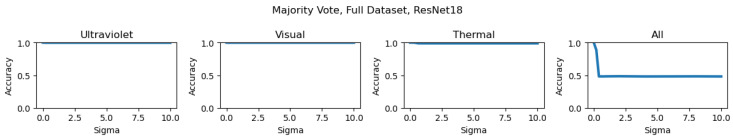
Majority voting results for the Full Dataset, showing the relationship between noise and model performance. Camera labels (Ultraviolet, Visual, Thermal, and All) show the input data to which noise was added.

**Figure 13:**
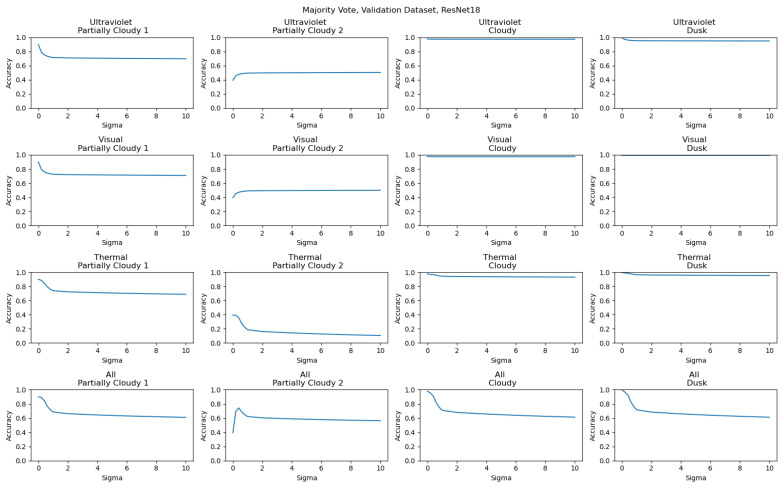
Majority voting results for the Validation Dataset, showing the relationship between noise and model performance. Camera labels (Ultraviolet, Visual, Thermal, and All) show the input data to which noise was added. The secondary labels show the validation dataset used.

**Figure 14:**
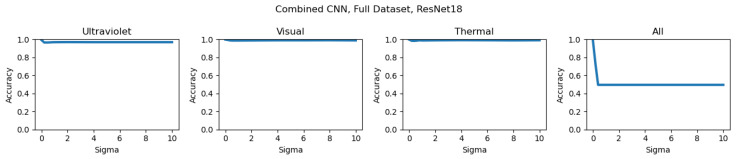
Combined CNN results for the Full Dataset, showing the relationship between noise and model performance. Camera labels (Ultraviolet, Visual, Thermal, All) show the input data to which noise was added.

**Figure 15:**
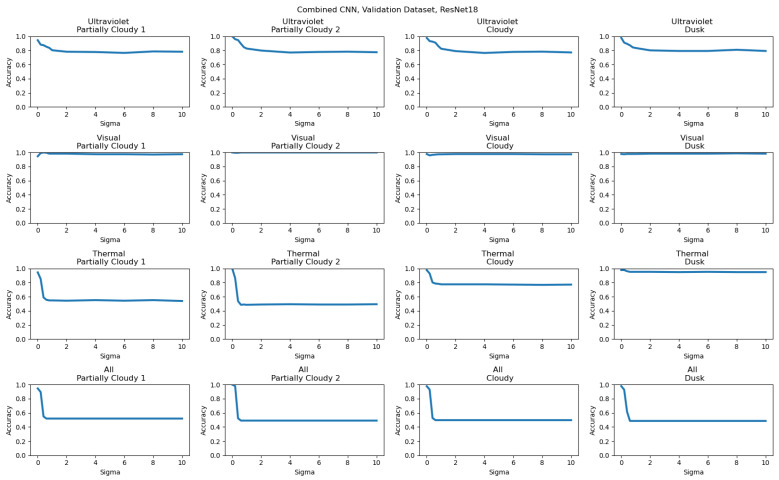
Combined CNN results for the Validation Dataset, showing the relationship between noise and model performance. Camera labels (Ultraviolet, Visual, Thermal, and All) show the input data to which noise was added. The secondary labels show the validation dataset used.

To better characterize the effects of data quality on ensemble models at different levels of input noise, additional results have been included showing the accuracy results of both the Full and Validation Datasets across different ensemble methods. The majority voting results for the Full Dataset are included in Figure 12, and for the Validation Dataset are included in Figure 13. The combined CNN results for the Full Dataset are shown in Figure 14, and for the Validation Dataset in shown in Figure 15. In every example, Gaussian noise is added with increasing sigma values to either one-input cameras (for the labels Ultraviolet, Visual, and Thermal) or equally to all inputs. For the Full Dataset results, significant changes are only seen when all inputs have noise added, as useful data being added from each input prevents any input from being too strongly weighted. In contrast, significant differences can be seen with the Validation Dataset, with the Thermal results being the strongest, as is consistent with the detailed individual results.

## 5. Discussion

Low-cost custom cameras can be successfully utilized for image classification with custom data; however, several factors relating to dataset quality can impact the effectiveness of this approach. An effective custom dataset must be varied enough to provide information regarding all the possible conditions that could affect the result, and the differences between those conditions may not always be intuitive. Testing on separate validation datasets can help improve the assessment process, providing insight into what conditions a model may not be adequately trained on or may be producing unexpected results. Model selection is also an important consideration, as different models or other choices, such as the use of transfer learning, can improve the classification accuracy even on more difficult data. Integration of cameras with different spectral ranges, which may behave differently in different conditions, can also help address these unknowns and improve overall performance, even without specializing fusion approaches for individual camera inputs.

### 5.1. Custom Dataset Practicality

Regardless of the approach to deep learning, data acquisition will likely be one of the most challenging components of the design. Locating a publicly available dataset that matches the needs of the application is a common approach, and, if possible, can significantly reduce the technical overhead required. However, the process of reviewing, cleaning, formatting, and otherwise adapting a dataset to a specific application can be a significant time sink, even without privacy or legal considerations that may be involved in acquiring access to a dataset. A custom dataset has the advantage of removing several of these potentially complicating factors; however, it can significantly increase the upfront work. The conditions needed for all classes, such as the generation of an electrical arc, may not be feasible to replicate in all cases, and if so, the amount of data needed for a deep learning model may be very significant. Approaches of data modeling and data augmentation may be able to make custom data more practical, though as was shown in this research, any augmented dataset would have to effectively represent the full variety of realistic conditions to be entirely effective. Additionally, while data collection can be automated, and ideally integrated into operational tasks, the technical overhead of implementing such an approach would have to be considered as part of a cost-saving approach.

However, for custom data the questions of ownership and legality of a custom dataset in most cases would be less complicated, as would access issues for future implementations of the data. A dataset collected using the same sensors as an application also has the advantage of reducing potential unknowns that could emerge in unpredictable ways from trained models. The results shown in this dataset, and in the validation dataset labeled Partially Sunny 2, show the potential for misclassification due to lack of data containing a specific angle of reflected light. In a system with fewer knowns and less user control, such as a publicly available dataset being applied to somewhat different applications, hidden variations in the data could be extremely difficult to identify. Creation of a custom dataset allows for significantly more control of the contents of the dataset, and the creation of validation datasets to assess the behavior of the models that can be verified by subject matter experts involved in the specific application. In cases where greater specific knowledge or greater trust in the sensor system is needed, these values may make custom data a viable option in spite of the technical overhead requirements.

### 5.2. Deep Learning Model Selection

The comparison between the Full and Validation dataset shows that the selection of model is more important for a more challenging problem. Of the models compared here, ResNet18 and VGGNet11 were in a majority of cases the models with the highest and most consistent classification accuracy. Of the ensemble models, even a simple deep learning-based ensemble, in the form of the combined CNNs, had significant improvement in most cases over many of the individual models. However, unlike an ensemble where weights are applied manually, such as the weighted majority vote compared in this paper, the weights applied by the training process are not transparent, and cannot be determined without testing. The noise tests show that in cases in which some inputs are more reliable than others, the models will likely become more reliant on certain inputs for those cases. While this outcome is reasonable based on other evidence and consistent with the weights chosen for the weighted majority vote, it could cause unpredictable behavior if unknown when assessing results, especially if some other validation mechanism is not successful in identifying damaged equipment.

### 5.3. Implementation of a Custom Sensor

This paper analyzes an implementation of a low-cost, custom sensor to the application of arc detection, using custom data. As is described above, the collection of custom data is a trade-off in technical overhead, and the practicality of the approach will depend on the limitations of a specific project. Collecting data and implementing low-cost equipment can have a high upfront cost in technical overhead, which will not be practical for all applications. Other applications may have strict budgets for the purchasing of new equipment over time, or may have more resources up front and reduced resources in the long term, making lower-cost replacements more economical than a faster or simpler implementation. Reduced equipment spending specifically was of interest to several of the electrical utilities interviewed in the course of this work, and the practicalities of one approach over another will depend on a variety of factors in addition to the technical requirements. The difficulty of the data collection will also depend on the application. If the collection of custom data is a fully isolated task, then it will likely have significant technical overhead. If relevant data instead can be collected in the process of maintenance, then building a library of data over time may be more feasible.

The findings of this paper show a clear correlation between classification quality and the quality of the training data, which is well known in machine learning. The results also show that the conditions that can result in lower quality data may not always be obvious, and can require domain knowledge as well as careful testing of datasets against variables. All training datasets used in this research contained a balance of data in direct sunlight as well as other light conditions. However, the Validation Dataset lacked sufficient samples of sunlight in which the source of light was behind the camera, particularly as in the dataset Partially Sunny 2. While potentially not a significant difference to human eyes, it caused almost 0% classification accuracies for the visual data, which could be entirely corrected by introducing similar samples into the training dataset. Custom implementations of such sensor systems will need to be aware of this, and can use domain knowledge to construct test cases and evaluate data carefully against a variety of conditions to mitigate the risk. The results of this paper do show that CNNs are powerful tools for classification even with lower-cost equipment, as long as they are trained on high quality, relevant data. The results also show that more difficult cases can perform at least somewhat better with larger, more complex models. Model choice also contains trade-offs in training and run-time, meaning a very representative dataset, while requiring more overhead to build, can also improve efficiency in other contexts.

Finally, in the case of ensemble models, the noise testing shows that models can become weighted in ways that may not be transparent to a user, depending on the ensemble implementation used. If this is a problem for a given application, additional test systems to validate the quality of the input data could reduce the risk of biased results. For arcing detection specifically, a false positive could be an annoyance, but a false negative could cause a significant fire risk. Quality assurance systems such as the validation of a detection system on a known target could improve the prediction quality and improve trust in a detection system. An implementation could also consider using multiple inputs of the same sensors, as even multiple lower-cost sensors can be less expensive than one higher quality sensor. As with the data collection approach, the more practical approach will vary with the constraints of the implementation.

Overall, the sensor solution examined in this paper will be most useful for projects with limitations on equipment budget, expectations of equipment damage requiring easy replacement, and availability for more upfront work to overcome the technical overhead. Aspects of this approach can also benefit applications in which the classification target is specific and cannot easily and accurately be found in public datasets.

### 5.4. Limitations and Future Work

Aspects of this work show that the integration of new data into a training dataset can significantly correct model performance. This work was limited to the analysis of different iterations of one set of collected data. An evaluation of a process identifying weaknesses within a training dataset, identifying the source and updating the training data accordingly would further improve functionality of the approach, and could be a direction for future work. Additionally, a greater use of data augmentation in combination with improved analysis of the dataset could reduce misclassifications, and reduce the potential overdependence of ensemble models on certain inputs if that behavior should be undesirable.

## 6. Conclusions

In this paper, we examine approaches to utilizing CNN-based image classification trained on a custom dataset for the implementation of a custom, low-cost camera system for electrical arc detection. For a very representative dataset, the approach was successful for the arc source and cameras used; however, the challenge of effectively collecting fully representative data, and approaches to mitigating the associated risks, is also examined in more depth. The effectiveness of detecting a light source, such as an electrical arc, optically in outdoor conditions will vary in difficulty relative the level of ambient light, and the challenge represented by that variability will become more significant in data with greater variation from the training data. Careful selection of models and model parameters, as well as the use of ensemble models as a customizable data fusion approach can help mitigate these risks, potentially even in the event of partial sensor failure. While building and evaluating custom datasets presents its own challenges compared to publicly available datasets, machine learning effectiveness can depend heavily on the quality of input data, especially for more niche applications. Custom data can make even lower-cost equipment effective as long as the representativeness of that data has been carefully evaluated to be of good quality and representative of realistic use cases. Additionally, integration of multiple data inputs that may not have the same failure cases can help offset variability within the data and improve overall performance. Awareness of those edge cases, along with validation of sensor quality, can be used to mitigate risks of partial sensor failure. While custom data can require more upfront work than alternatives, careful construction of custom data can make a low-cost, adaptable multispectral sensor an effective option.

## Figures and Tables

**Table 1:** The class composition of each of the custom datasets collected.

Dataset	On	Off	Total
Train	2418	2406	4824
Partially Cloudy 1	115	124	239
Partially Cloudy 2	127	122	249
Cloudy	135	133	268
Dusk	122	115	237

**Table 2:** Validation data weather conditions, with conditions taken from NOAA data for location of data collection, and weather conditions visually verified. Times shown are from timestamps of the spectrometer data, which was taken immediately before collection of each validation dataset.

Validation Dataset	Temperature	Humidity	Weather Condition	Time (EST)
Partially Cloudy 1	41 °F	76%	Partially Cloudy	12:18 p.m.
Partially Cloudy 2	50 °F	58%	Partially Cloudy	12:19 p.m.
Cloudy	50 °F	58%	Cloudy	12:06 p.m.
Dusk	38 °F	41%	Cloudy	5:09 p.m.

**Table 3:** Accuracy results for 5-fold cross-validation of all the data collected, including all of the original validation data into the cross-validation datasets, and randomizing the training and validation data over all collected data. The results show the means and standard deviations for the five cross-validation runs.

Cross-Validation Full Dataset Metrics			
	Accuracy		
Model	Ultraviolet	Visual	Thermal
Transfer Learning			
AlexNet	0.865 ± 0.198	1.0 ± 0	1.0 ± 0
ResNet18	0.995 ± 0.003	1.0 ± 0	1.0 ± 0.001
VGGNet11	0.989 ± 0.002	1.0 ± 0	1.0 ± 0
No Transfer Learning			
AlexNet	0.880 ± 0.13	0.982 ± 0.036	0.995 ± 0.003
ResNet18	0.987 ± 0.011	1.0 ± 0	0.998 ± 0.003
VGGNet11	0.968 ± 0.022	1.0 ± 0.001	0.998 ± 0.003
	F1		
Transfer Learning			
AlexNet	0.768 ± 0.411	1.0 ± 0	1.0 ± 0
ResNet18	0.995 ± 0.004	1.0 ± 0	1.0 ± 0.001
VGGNet11	0.989 ± 0.003	1.0 ± 0	1.0 ± 0
No Transfer Learning			
AlexNet	0.883 ± 0.121	0.981 ± 0.039	0.995 ± 0.003
ResNet18	0.986 ± 0.012	1.0 ± 0	0.998 ± 0.003
VGGNet11	0.967 ± 0.024	1.0 ± 0.001	0.998 ± 0.004

**Table 4:** Accuracy results for data from each separate camera, across the primary model parameters compared, for each of the validation datasets, and the combined result for all four validation datasets. This data represents the mean of five different models with randomized weights, with standard deviations to show the stability of the models.

Validation Dataset Accuracy					
	PC1	PC2	Cloudy	Dusk	All
Ultraviolet					
Transfer Learning					
AlexNet	0.811 ± 0.039	0.13 ± 0.05	0.829 ± 0.032	0.959	0.68 ± 0.021
ResNet18	0.501 ± 0.067	0.308 ± 0.046	0.925 ± 0.012	0.948 ± 0.042	0.674 ± 0.024
VGGNet11	0.63 ± 0.065	0.392 ± 0.105	0.924 ± 0.008	0.966 ± 0.0	0.73 ± 0.027
No Transfer Learning					
AlexNet	0.567 ± 0.034	0.361 ± 0.146	0.607 ± 0.119	0.63 ± 0.189	0.541 ± 0.074
ResNet18	0.804 ± 0.088	0.267 ± 0.154	0.898 ± 0.007	0.959 ± 0.006	0.732 ± 0.034
VGGNet11	0.562 ± 0.075	0.361 ± 0.154	0.661 ± 0.147	0.879 ± 0.084	0.614 ± 0.03
Visual					
Transfer Learning					
AlexNet	0.527 ± 0.137	0.127 ± 0.052	0.572 ± 0.062	0.745 ± 0.03	0.491 ± 0.046
ResNet18	0.582 ± 0.181	0.018 ± 0.019	0.695 ± 0.099	0.698 ± 0.178	0.498
VGGNet11	0.484 ± 0.176	0.308 ± 0.089	0.633 ± 0.051	0.718 ± 0.075	0.536 ± 0.048
No Transfer Learning					
AlexNet	0.428 ± 0.154	0.009 ± 0.02	0.559 ± 0.021	0.719 ± 0.049	0.0428
ResNet18	0.719 ± 0.214	0.111 ± 0.119	0.727 ± 0.138	0.557 ± 0.031	0.53 ± 0.066
VGGNet11	0.537 ± 0.07	0.004 ± 0.009	0.199 ± 0.037	0.544 ± 0.064	0.314 ± 0.024
Thermal					
Transfer Learning					
AlexNet	0.948 ± 0.013	1.0 ± 0.0	0.924 ± 0.022	0.964	0.958 ± 0.009
ResNet18	0.958 ± 0.031	1.0 ± 0.0	0.967 ± 0.011	0.962 ± 0.013	0.972 ± 0.012
VGGNet11	0.948 ± 0.033	0.999 ± 0.002	0.972 ± 0.021	0.957 ± 0.005	0.97 ± 0.008
No Transfer Learning					
AlexNet	0.968 ± 0.02	0.998 ± 0.004	0.693 ± 0.116	0.844 ± 0.09	0.872 ± 0.056
ResNet18	0.935 ± 0.02	1.0 ± 0.0	0.887 ± 0.114	0.904 ± 0.076	0.931 ± 0.045
VGGNet11	0.978 ± 0.016	1.0 ± 0.0	0.925 ± 0.012	0.934 ± 0.011	0.969 ± 0.005

**Table 5:** Accuracy values for a majority vote done with the full dataset.

Majority Vote-Full Dataset		
AlexNet	ResNet18	VGGNet11
1.0	1.0	1.0

**Table 6:** Accuracy results for majority voting ensemble models for the Validation Dataset, including both a simple and a weighted majority vote.

Majority Vote-Validation Dataset				
	Partially Cloudy 1	Partially Cloudy 2	Cloudy	Dusk
Separate Models				
AlexNet	0.778	0.193	0.899	0.992
ResNet18	0.9	0.394	0.978	0.996
VGGNet11	0.695	0.442	0.981	0.987
All Models Weighted				
All	0.971	0.55	0.996	0.996

**Table 7:** Accuracy values for combined CNN models with the Full Dataset.

Combined CNN-Full Dataset		
AlexNet	ResNet18	VGGNet11
0.997	0.998	0.992

**Table 8:** Accuracy results for combined CNN models for the Validation Dataset.

Combined CNN				
	Partially Cloudy 1	Partially Cloudy 2	Cloudy	Dusk
AlexNet	0.954	0.932	0.933	0.975
ResNet18	0.946	1	0.978	0.979
VGGNet	0.937	0.996	0.974	0.97

**Table 9:** Majority vote results with noise added to each of the camera inputs. The highest accuracy of each validation dataset, for each camera, has been bolded.

Majority Vote-Validation Dataset				
	Partially Cloudy 1	Partially Cloudy 2	Cloudy	Dusk
Separate Models				
**Ultraviolet Camera Noise**				
AlexNet	0.649	**0.582**	0.664	0.911
ResNet18	**0.678**	0.514	**0.974**	**0.945**
VGGNet	0.569	0.51	0.713	0.814
**Visual Camera Noise**				
AlexNet	0.866	0.534	0.888	0.979
ResNet18	**0.690**	0.510	**0.974**	**0.996**
VGGNet	0.653	**0.610**	0.963	0.987
**Thermal Camera Noise**				
AlexNet	0.527	0.024	0.634	0.899
ResNet18	**0.628**	0.008	0.918	**0.941**
VGGNet	0.536	**0.197**	0.937	0.920
**All Models Weighted**				
UV Noise	0.527	0.024	0.634	0.899
Visual Noise	**0.628**	0.008	0.918	**0.941**
Thermal Noise	0.536	**0.197**	**0.937**	0.920

**Table 10:** Combined CNN results with noise added to each of the camera inputs. The highest accuracy of each validation dataset, for each camera, has been bolded.

Combined CNN				
	Partially Cloudy 1	Partially Cloudy 2	Cloudy	Dusk
**Ultraviolet Camera Noise**				
AlexNet	**0.954**	0.924	0.937	0.949
ResNet18	0.774	0.779	0.784	0.797
VGGNet	0.937	**1.0**	**0.974**	**0.966**
**Visual Camera Noise**				
AlexNet	0.962	**1.0**	0.813	0.966
ResNet18	**0.975**	**1.0**	**0.981**	**0.983**
VGGNet	0.950	**1.0**	0.963	0.975
**Thermal Camera Noise**				
AlexNet	0.519	0.490	0.619	0.713
ResNet18	**0.544**	**0.494**	**0.769**	**0.949**
VGGNet	0.523	0.474	0.496	0.540

## Data Availability

The original data presented in the study are openly available in IEEE DataPort at https://ieee-dataport.org//documents/custom-multispectral-camera-recorded-electrical-arcing (accessed on 7 February 2025).
